# Low-dose real-time X-ray imaging with nontoxic double perovskite scintillators

**DOI:** 10.1038/s41377-020-00353-0

**Published:** 2020-06-30

**Authors:** Wenjuan Zhu, Wenbo Ma, Yirong Su, Zeng Chen, Xinya Chen, Yaoguang Ma, Lizhong Bai, Wenge Xiao, Tianyu Liu, Haiming Zhu, Xiaofeng Liu, Huafeng Liu, Xu Liu, Yang (Michael) Yang

**Affiliations:** 1 State Key Laboratory of Modern Optical Instrumentation, College of Optical Science and Engineering, International Research Center for Advanced Photonics, Zhejiang University, Hangzhou, Zhejiang China; 2 Center for Chemistry of High-Performance & Novel Materials, department of Chemistry, Zhejiang University, Hangzhou, Zhejiang China

**Keywords:** Optical materials and structures, Lasers, LEDs and light sources

## Abstract

X-rays are widely used in probing inside information nondestructively, enabling broad applications in the medical radiography and electronic industries. X-ray imaging based on emerging lead halide perovskite scintillators has received extensive attention recently. However, the strong self-absorption, relatively low light yield and lead toxicity of these perovskites restrict their practical applications. Here, we report a series of nontoxic double-perovskite scintillators of Cs_2_Ag_0.6_Na_0.4_In_1-y_Bi_y_Cl_6_. By controlling the content of the heavy atom Bi^3+^, the X-ray absorption coefficient, radiative emission efficiency, light yield and light decay were manipulated to maximise the scintillator performance. A light yield of up to 39,000 ± 7000 photons/MeV for Cs_2_Ag_0.6_Na_0.4_In_0.85_Bi_0.15_Cl_6_ was obtained, which is much higher than that for the previously reported lead halide perovskite colloidal CsPbBr_3_ (21,000 photons/MeV). The large Stokes shift between the radioluminescence (RL) and absorption spectra benefiting from self-trapped excitons (STEs) led to a negligible self-absorption effect. Given the high light output and fast light decay of this scintillator, static X-ray imaging was attained under an extremely low dose of ∼1 μGy_air_, and dynamic X-ray imaging of finger bending without a ghosting effect was demonstrated under a low-dose rate of 47.2 μGy_air_ s^−1^. After thermal treatment at 85 °C for 50 h followed by X-ray irradiation for 50 h in ambient air, the scintillator performance in terms of the RL intensity and X-ray image quality remained almost unchanged. Our results shed light on exploring highly competitive scintillators beyond the scope of lead halide perovskites, not only for avoiding toxicity but also for better performance.

## X-ray imaging: Better crystals to convert X-rays into light

Low-dose X-ray imaging, which relies on scintillators to convert X-ray photons into readily detectable photons of light, has been improved using new varieties of scintillators composed of materials known as double perovskites. Yang Yang and colleagues at Zhejiang University in China made their scintillators from nontoxic elements including caesium, silver, sodium, indium, bismuth and chloride ions. They outperform existing lead halide perovskite scintillators due to greatly increased light-yield, heat stability and low toxicity. Unlike many alternatives, they do not contain toxic lead ions. The researchers maximised the light-yielding efficiency by controlling the content of bismuth ions (Bi^3+^) in the double perovskite crystals. They demonstrated the potential of their material by achieving exceptionally low-dose static X-ray images and real-time dynamic images of finger bending. The researchers see great scope for further improvements by exploring other types of lead-free perovskites and perovskite derivatives.

## Introduction

X-ray imaging has been actively utilised in the fields of industrial material inspection, medical diagnosis and scientific research^1–10^. Low-dose irradiation, high stability and high spatial resolution are generally regarded as the most important characteristics for X-ray imaging^11,12^. Current X-ray imaging systems mostly rely on scintillators that are capable of converting X-ray photons into visible photons that are then detected by a photodiode array^13–15^. Conventional scintillators, such as thallium-doped caesium iodide (CsI:Tl)^16,17^ and cerium-doped lutetium−aluminium garnet (LuAG:Ce)^18^, usually require expensive and time-consuming synthesis, which poses a major challenge for device processability. Unlike conventional scintillator materials, the emerging lead halide perovskites for X-ray detectors are starting to show attractive merits of facile fabrication, fast response and good spatial resolution^19–26^. However, the relatively low X-ray light yield, lead toxicity^27–29^ and instability greatly limit their applications in high-end X-ray imaging featuring low-dose exposure, hazard-free manufacturing, real-time monitoring and robustness.

Fortunately, previous efforts have discovered many efficient lead-free emitters, e.g., double-perovskite^30–32^, copper^33–35^ and bismuth (Bi)^36–38^-based metal halides, which hold potential for X-ray scintillators. Very recently, Rb_2_CuBr_3_^33^ and Cs_2_NaTbCl_6_^39^ were shown to be scintillators with high light yield. However, the long decay time and strong afterglow impede their use in realising high contrast X-ray imaging, especially for X-ray computed tomography (CT). Another limitation of Rb_2_CuBr_3_ is that its emission wavelength is in the blue region, which does not match the peak response of the common camera. Hence, the development of nontoxic halide scintillators with merits of high light yield, fast light decay and well-matched emission wavelength remains a challenge. In this article, a series of nontoxic double perovskites of Cs_2_Ag_0.6_Na_0.4_In_1-y_Bi_y_Cl_6_ single crystals with variable Bi^3+^ content were prepared. The introduction of a moderate amount of Bi^3+^ not only improves the radioluminescence (RL) output but also accelerates the radiative recombination, leading to a high scintillator light yield of 39,000 ± 7000 photons/MeV and fast light decay for Cs_2_Ag_0.6_Na_0.4_In_0.85_Bi_0.15_Cl_6_. The nontoxic scintillator delivers long-term stability under continuous thermal treatment and X-ray irradiation in ambient air. With a Cs_2_Ag_0.6_Na_0.4_In_0.85_Bi_0.15_Cl_6_ wafer as the scintillator, high-quality static and dynamic images of different objects were obtained under low-dose X-ray irradiation.

## Results

Commonly, the double-perovskite structure Cs_2_B^I^B^III^Cl_6_ is regarded as a homologue of the ABX_3_-type perovskite, in which the B sites are replaced by equal amounts of monovalent and trivalent cations^40^. Herein, B^I^ is occupied by Ag^+^ and Na^+^ with different alloying ratios. By means of Na^+^ doping in Cs_2_AgInCl_6_, the parity-forbidden transition is partly broken, and the electronic dimensionality is reduced as well^31^. Therefore, bright near-white light emission via radiative recombination of self-trapped excitons (STEs) of Cs_2_Ag_1-*x*_Na_x_InCl_6_ (*x* = 0.2, 0.4, 0.6, 0.8) single crystals is obtained. The optimised Na^+^ content *x* is determined to be 0.4, for which the photoluminescence quantum yield (PLQY) reaches 43% (Supplementary Fig. S1).

**Fig. 1:**
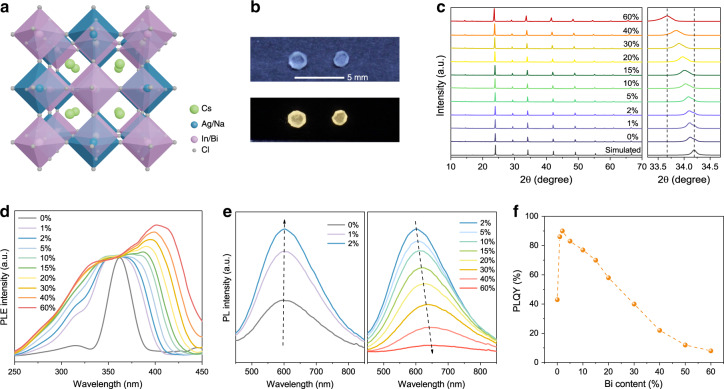
Crystal structure and photoluminescence (PL) characterisation of Cs_2_Ag_0.6_Na_0.4_In_1-y_Bi_y_Cl_6_. **a** Crystal structure of the double perovskite. **b** Photographs of Cs_2_Ag_0.6_Na_0.4_In_0.85_Bi_0.15_Cl_6_ single crystals under daylight and UV light excitation. **c** XRD patterns (left) and selected diffraction peaks near 34° (right) of Cs_2_Ag_0.6_Na_0.4_In_1-y_Bi_y_Cl_6_ with different Bi^3+^ contents. **d** Photoluminescence excitation (PLE) spectra and **e** PL spectra of Cs_2_Ag_0.6_Na_0.4_In_1-y_Bi_y_Cl_6_ powder with different Bi^3+^ contents. **f** Photoluminescence quantum yield (PLQY) of Cs_2_Ag_0.6_Na_0.4_In_1-y_Bi_y_Cl_6_ powder with various Bi^3+^ contents

Bi is an earth-abundant and green element, and it has an even larger atomic number than the widely used heavy X-ray absorbing elements of Pb and TI; hence, we introduced partial Bi^3+^ to replace In^3+^, initially for the purpose of increasing the X-ray absorption efficiency^41,42^. The powder X-ray diffraction (PXRD) patterns of a series of Cs_2_Ag_0.6_Na_0.4_In_1-y_Bi_y_Cl_6_ (Fig. 1c) samples confirm that the pure double-perovskite phase is the same as Cs_2_AgInCl_6_, which belongs to space group *Fm-3m* with a face-centred cubic structure. It is worth mentioning that the PXRD peaks shift to a lower diffraction angle with increasing Bi^3+^ content due to the larger ionic radius of Bi^3+^ (103 pm) than that of In^3+^ (80 pm), which can be clearly observed in the zoomed-in figure (right side of Fig. 1c). In addition, the scanning electron microscopy–energy-dispersive spectrometry (SEM–EDS) results (Supplementary Table S2) of six averaged random spots on the testing sample demonstrate that the final chemical compositions of Cs_2_Ag_0.6_Na_0.4_In_1-y_Bi_y_Cl_6_ agree with the designed ratios. All the above analyses suggest that alloyed compounds of Cs_2_Ag_0.6_Na_0.4_In_1-y_Bi_y_Cl_6_ are successfully synthesised, whose chemical components change regularly, depending on the Bi/In ratio.

Interestingly, in addition to the expected enhancement of the X-ray absorption, the luminescence properties of the alloyed double perovskites can also be tuned by Bi^3+^ alloying. The photoluminescence excitation (PLE) peak near 362 nm becomes more distinct and broader as the Bi^3+^ content increases (Fig. 1d), which is consistent with the steady-state absorption spectra (Supplementary Fig. S2). To evaluate the absorption band tail width of Cs_2_Ag_0.6_Na_0.4_In_1-y_Bi_y_Cl_6_, we calculated the Urbach energy (*E*_U_) from the plotted straight fitting lines, and the corresponding values are given (Supplementary Fig. S2). Compared with Cs_2_Ag_0.6_Na_0.4_InCl_6_, the *E*_U_ of the Bi^3+^-doped perovskites significantly decreases, demonstrating that the band tail can be effectively suppressed because of further breaking of the parity-forbidden transition by the incorporated Bi^3+^^43–45^. When introducing more Bi^3+^, the absorption peak at 362 nm widens gradually and becomes less clear, and its intensity increases and is finally saturated in agreement with the PLE spectra, suggesting multiple absorption states that are widely distributed. The incorporated Bi^3+^ contributes to the valence-band structure by introducing shallow states right above the valence-band maximum of Cs_2_Ag_0.6_Na_0.4_In_1-y_Bi_y_Cl_6_^31^ and forming a hyperfine energy level^30^, leading to broad absorption and excitation. Meanwhile, the absorption edges redshift as the Bi/In ratio increases, and the bandgap (*E*_g_) values were calculated from Tauc plots (Supplementary Fig. S3), demonstrating that the bandgap is monotonically decreasing^46^. Figure 1e shows the PL spectra of Cs_2_Ag_0.6_Na_0.4_In_1-y_Bi_y_Cl_6_ as a function of Bi^3+^ content. The PL intensity rapidly intensifies when the Bi^3+^ concentration increases from 0 to 2% and then weakens with further doping of Bi^3+^, obtaining the highest PLQY of 90% (Fig. 1f). Meanwhile, the PL peak redshifts from 605 nm to 652 nm due to the gradually narrowed bandgap caused by the Bi^3+^ substitution of In^3+^, in agreement with the absorption spectra. Apparently, the PL and absorption spectra overlap for this double-perovskite system is negligible. The large Stokes shift avoids the self-absorption effect that is detrimental to the output of scintillation light^14^. Both the outstanding PL efficiency and negligible self-absorption of Cs_2_Ag_0.6_Na_0.4_In_1-y_Bi_y_Cl_6_ imply that these materials have the potential to be good scintillators.

The light decay time is another figure of merit for scintillators. The TRPL of Cs_2_Ag_0.6_Na_0.4_In_1-y_Bi_y_Cl_6_ was obtained by the TCSPC method pumped with a femtosecond laser (400 nm, <300 fs, 1 MHz). As shown in Supplementary Fig. S4, the decay curve of Cs_2_Ag_0.6_Na_0.4_InCl_6_ can be fitted by a biexponential function, giving a fast decay process (~1 ns) and a slower decay process (2.8 μs), which is assigned to the forbidden STE emission^30^. To investigate the origin of the fast decay process, the TRPL of the STE-emission band (we used a filter to selectively collect the light signal from 700 nm to 800 nm) was monitored, and it still showed distinct fast decay (Supplementary Fig. S5), excluding the possibility of band-to-band radiative recombination. This fast component gradually vanishes with the incorporation of Bi^3+^. Hence, the fast decay process (~1 ns) may be assigned to the defect trapping process^30,45^ since Bi^3+^ can passivate defects^31^. In addition, when collecting the TRPL with different time resolutions (4 ps/16 ps/512 ps), the fast decay process becomes unobvious when the time resolution is 512 ps (Supplementary Fig. S5), indicating that this ultrafast process cannot be detected with low time resolution. With increasing Bi^3+^ content, the lifetime of the slow component gradually shortens to the nanosecond level, which is superior to that of the current commercial CsI:Tl^16^, demonstrating the great potential for dynamic real-time X-ray imaging. We reason that the breaking of the parity-forbidden transition induced by Bi^3+^ doping results in improved radiative recombination kinetics; therefore, the lifetime becomes shorter.

**Fig. 2:**
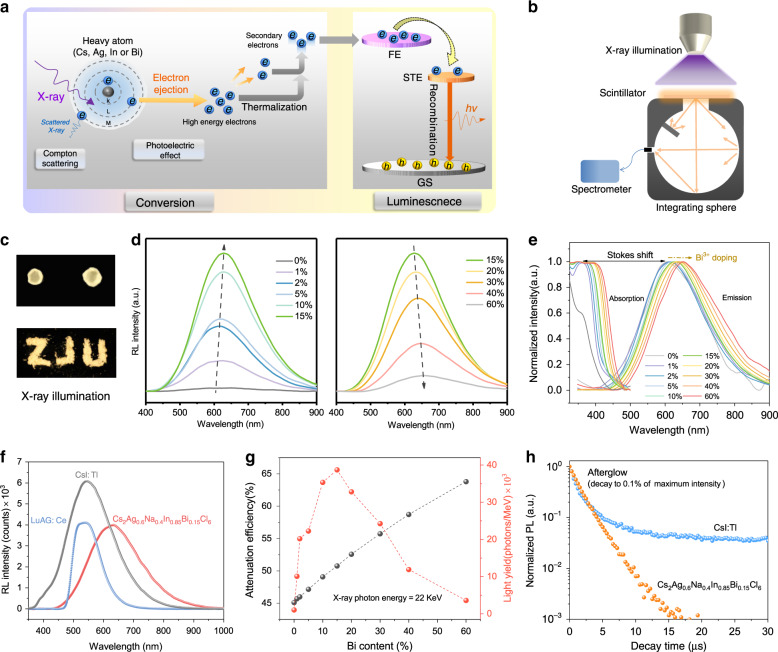
Radioluminescence (RL) characterisation of Cs_2_Ag_0.6_Na_0.4_In_1-y_Bi_y_Cl_6_ scintillators. **a** Proposed mechanism of X-ray scintillation in a lead-free halide double-perovskite scintillator. **b** Schematic of RL spectra measurement using an integrating sphere and a spectrometer with a fixed X-ray source-to-sample distance. **c** Photographs of Cs_2_Ag_0.6_Na_0.4_In_0.85_Bi_0.15_Cl_6_ single crystals and powder under X-ray illumination (dose rate: 189 μGy_air_ s^−1^, voltage: 50 kV). **d** RL spectra of Cs_2_Ag_0.6_Na_0.4_In_1-y_Bi_y_Cl_6_ powder with different Bi^3+^ contents under X-ray excitation with a dose rate of 189 μGy_air_ s^−1^ at a voltage of 50 kV. **e** Stokes shift of Cs_2_Ag_0.6_Na_0.4_In_1-y_Bi_y_Cl_6_ with different Bi^3+^ contents. **f** RL spectra of Cs_2_Ag_0.6_Na_0.4_In_0.85_Bi_0.15_Cl_6_, LuAG:Ce and CsI:Tl wafers (dose rate: 189 μGy_air_ s^−1^, voltage: 50 kV). **g** Attenuation efficiency and light yield of Cs_2_Ag_0.6_Na_0.4_In_1-y_Bi_y_Cl_6_ versus Bi^3+^ content. **h** Afterglow curves of Cs_2_Ag_0.6_Na_0.4_In_0.85_Bi_0.15_Cl_6_ and CsI:Tl

To assess the scintillation property, a series of Cs_2_Ag_0.6_Na_0.4_In_1-y_Bi_y_Cl_6_ were tested under X-ray illumination. Figure 2a shows a plausible mechanism of X-ray scintillation in the lead-free halide double perovskites. The radiation energy is first absorbed by the heavy atoms of the double perovskites mainly through the photoelectric effect and inelastic Compton scattering, ejecting massive hot electrons; then, these electrons thermalise on an ultrafast timescale and are captured by luminescent centres^47,48^. The high PLQY of our double-perovskite ensures that once electrons transfer to the recombination centre, the ultimate radiative emission is very efficient. To accurately measure the RL, equimolecular Cs_2_Ag_0.6_Na_0.4_In_1-y_Bi_y_Cl_6_ powder with various Bi ratios were compressed into compact wafers, and then, these wafers were closely attached to the circular window of an integrating sphere with a fixed distance to the X-ray source. The corresponding RL spectra were recorded by a fibre-coupled spectrometer (Fig. 2b). Figure 2c shows photographs of Cs_2_Ag_0.6_Na_0.4_In_0.85_Bi_0.15_Cl_6_ SCs and powder under X-ray excitation, yielding strong and uniform light-yellow emission. Figure 2d shows that the scintillation output has a nonmonotonic relation with the Bi ratio. It first increases, reaches the peak intensity at 15% Bi^3+^, and then drops as the Bi^3+^ content further increases, which is mainly attributed to the collective effect of the X-ray absorption efficiency (Fig. 2g) and radiative emission efficiency that can be reflected by the PLQY (Fig. 1f). As we anticipated, the calculated X-ray attenuation efficiency of Cs_2_Ag_0.6_Na_0.4_In_1-y_Bi_y_Cl_6_ enhances monotonically with Bi^3+^ doping because of the large atomic number of Bi^3+^ (Fig. 2g). Herein, when the Bi^3+^ content is increased from 0 to 2%, both the PLQY and X-ray absorption of Cs_2_Ag_0.6_Na_0.4_In_1-y_Bi_y_Cl_6_ improve, leading to a rapid increase in the RL intensity. With further doping of Bi^3+^ from 2% to 15%, the near-linear improvement of the X-ray absorption dominates the slowly declining radiative emission efficiency, as manifested by the PLQY, resulting in continuous enhancement of the RL intensity. With Bi^3+^ doping greater than 15%, the contribution of rapidly decreased PLQY exceeds that of the increased X-ray absorption efficiency of Bi^3+^, and the overall RL intensity diminishes. The shapes of the RL spectra are consistent with those of the corresponding PL spectra, indicating that the last step of an X-ray scintillation event is the same as the PL process, that is, they both emit light through STEs. One unique feature of STE emission is that the absorption and RL peak have a large Stokes shift, leading to negligible self-absorption, while a Pb-based perovskite scintillator, e.g., CsPbBr_3_, has a very small Stokes shift due to its direct bandgap^4^. This large Stokes shift is generally favourable for any emitters but particularly desirable for scintillators since scintillators are very thick to ensure sufficient absorption of X-rays, but the scintillations are usually weak^14^. As shown in Supplementary Fig. S6, the integrated RL intensity of Cs_2_Ag_0.6_Na_0.4_In_0.85_Bi_0.15_Cl_6_ has a linear response to the X-ray dose rate, highlighting its suitability for X-ray contrast imaging.

Equivalent to the PLQY, the light yield (LY) of a scintillator is regarded as the internal X-ray-to-photon conversion efficiency, which can be measured as the ratio of the total emitted photon number to the absorbed X-ray energy^47^. Theoretically, the LY is governed by the relation LY = 10^6^ × SQ/(βE_g_), where β is a constant related to the host structure. As mentioned above, Eg slightly decreases with increasing Bi^3+^ content, which is a negligible factor for the LY in Cs_2_Ag_0.6_Na_0.4_In_1-y_Bi_y_Cl_6_. Hence, both the transfer efficiency S of hot-electron energy and the photoluminescence quantum yield Q (PLQY) govern the LY. In an attempt to quantify the RL light yield, we selected a commercial LuAG:Ce scintillator as a reference, whose light yield is 22,000 ± 4000 photons/MeV. To unify the absorbed X-ray energies of these two kinds of scintillator, the attenuation efficiencies of LuAG: Ce and Cs_2_Ag_0.6_Na_0.4_In_0.85_Bi_0.15_Cl_6_ as a function of thickness at an X-ray photon energy of 22 keV were calculated, since the major X-ray photon energy of our tube is 22 keV (Supplementary Fig. S7). Based on this relation curve, a Cs_2_Ag_0.6_Na_0.4_In_0.85_Bi_0.15_Cl_6_ wafer with a 0.4-mm thickness and a LuAG:Ce wafer with a 0.11-mm thickness were fabricated, and the corresponding RL spectra were recorded (Fig. 2f). In such circumstances, LuAG:Ce and Cs_2_Ag_0.6_Na_0.4_In_0.85_Bi_0.15_Cl_6_ have the same X-ray absorption cross sections, and therefore, the light output can be fairly compared. Finally, Cs_2_Ag_0.6_Na_0.4_In_0.85_Bi_0.15_Cl_6_ delivers a light yield of 39,000 ± 7000 photons per MeV, comparable with that of commercial CsI:Tl and much higher than that of the previously reported lead halide perovskite colloidal CsPbBr_3_ (21,000 photons/MeV)^3^. In addition, the light yields of Cs_2_Ag_0.6_Na_0.4_In_1-y_Bi_y_Cl_6_ for various Bi^3+^ contents are given in Fig. 2g, whose evolution trend corresponds well with the RL output intensity. It is noted that the trend of the scintillator light yield with the Bi^3+^ doping ratio is not exactly the same as that of the PLQY; Cs_2_Ag_0.6_Na_0.4_In_0.85_Bi_0.15_Cl_6_ has the highest scintillation light yield, while Cs_2_Ag_0.6_Na_0.4_In_0.98_Bi_0.02_Cl_6_ has the best PLQY. This highlights the fact that optimisation of the scintillator performance cannot always follow the PLQY results since the PLQY only reflects the efficiency of the last step of a scintillation event, that is, the radiative emission of the thermalized electrons. In addition to the PLQY, the transfer efficiency S of hot-electron energy also plays a decisive role in the LY. Owing to the high atomic number of Bi, we speculate that there is a tight radius of the hot-electron distribution around Bi^3+^ in Cs_2_Ag_0.6_Na_0.4_In_1-y_Bi_y_Cl_6_, which is beneficial to hot-electron energy transfer^48^. The LY value is maximised when the Bi^3+^ doping is 15% due to the collective effect of S and Q, and the SQ product is optimised under this circumstance. Another interesting discovery is that the optimised Cs_2_Ag_0.6_Na_0.4_In_0.85_Bi_0.15_Cl_6_ scintillator shows strong X-ray absorption efficiency from ~36 keV to 60 keV, which is the region for common medical digital radiography (Supplementary Fig. S8). Another important parameter for scintillators is the afterglow, which can reduce the signal-to-noise ratio (SNR) of the X-ray imaging. To obtain high contrast imaging without lag, it is always desirable to reduce the afterglow, especially for CT imaging. As shown in Fig. 2h, the luminance signal of Cs_2_Ag_0.6_Na_0.4_In_0.85_Bi_0.15_Cl_6_ decays to 0.1% at ~16 μs. This low afterglow significantly outperforms that of the previous Rb_2_CuBr_3_ (2.72‰ @ 20 ms)^33^ and is much lower than that of the widely used scintillator CsI:Tl (1.5% @ 3 ms), demonstrating the great potential of Cs_2_Ag_0.6_Na_0.4_In_0.85_Bi_0.15_Cl_6_ for real-time X-ray imaging and medical CT applications.

**Fig. 3:**
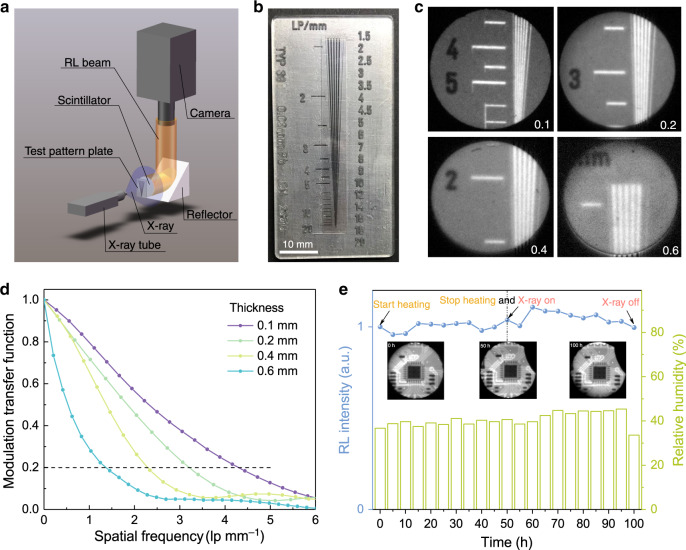
X-ray imaging based on Cs_2_Ag_0.6_Na_0.4_In_0.85_Bi_0.15_Cl_6_ scintillator wafers with different thicknesses. **a** Schematic of the X-ray imaging system. **b** Photograph of the standard X-ray test-pattern plate. **c** X-ray images of the test-pattern plate based on Cs_2_Ag_0.6_Na_0.4_In_0.85_Bi_0.15_Cl_6_ wafers with different thicknesses (dose rate: 189 μGy_air_ s^−1^, voltage: 50 kV). **d** Corresponding MTF curves of Cs_2_Ag_0.6_Na_0.4_In_0.85_Bi_0.15_Cl_6_ wafers with different thicknesses. **e** Integrated RL intensity of Cs_2_Ag_0.6_Na_0.4_In_0.85_Bi_0.15_Cl_6_ under thermal treatment for 50 h at 85 °C followed by X-ray illumination for another 50 h (dose rate: 12 μGy_air_ s^−1^, voltage: 50 kV). The moisture level was also recorded, and the RL was measured with an integrating sphere. The inset shows X-ray images of a circuit board acquired at three different stages (0 h, 50 h and 100 h) with a dose rate of 189 μGy_air_ s^−1^ at a voltage of 50 kV

To implement X-ray imaging with Cs_2_Ag_0.6_Na_0.4_In_0.85_Bi_0.15_Cl_6_ as a scintillator, a homemade optical system was built, as illustrated in Fig. 3a. Cs_2_Ag_0.6_Na_0.4_In_0.85_Bi_0.15_Cl_6_ wafers with different thicknesses (0.1 mm, 0.2 mm, 0.4 mm and 0.6 mm) were tested for X-ray imaging performance. The X-ray images of the standard test-pattern plate given in Fig. 3c demonstrate that the spatial resolution decreases with thickening wafer due to the increased optical crosstalk caused by light scattering. To quantify the spatial resolution, the modulation transfer function (MTF) was calculated using slanted-edge images^49^ (Supplementary Fig. S9). As shown in Fig. 3d, the spatial resolution (which is defined as the spatial frequency value at MTF = 0.2) is determined to be 4.3 lp mm^−1^, 3.2 lp mm^−1^, 2.3 lp mm^−1^ and 1.4 lp mm^−1^ for Cs_2_Ag_0.6_Na_0.4_In_0.85_Bi_0.15_Cl_6_ wafers with thicknesses of 0.1 mm, 0.2 mm, 0.4 mm and 0.6 mm, respectively, which are consistent with the values from the X-ray images of the standard test-pattern plate. The spatial resolution of the Cs_2_Ag_0.6_Na_0.4_In_0.85_Bi_0.15_Cl_6_ wafer with a 0.1-mm thickness is comparable with that of a Se direct-type X-ray imager (4.8 lp mm^−1^ at MTF = 0.2)^2,50^. There is no doubt that further decreasing the thickness of the scintillator wafer can help improve the spatial resolution of X-ray imaging, but a larger dose rate will be needed to produce a sufficient scintillating light signal when taking real-time imaging^51^. It is also worth mentioning that the spatial resolution can be further enhanced if the scintillator screen is closely attached to the CMOS panel since the optical crosstalk will be minimised in this case. However, with such an optical configuration, the field of view (FOV) is restricted by the size of the very expensive CMOS chip, while with our optical setup, we can view large objects such as human fingers with a 13.2-mm × 13.2-mm CMOS chip.

The environmental, thermal and X-ray radiation stability of the Cs_2_Ag_0.6_Na_0.4_In_0.85_Bi_0.15_Cl_6_ scintillator regarding the RL intensity and X-ray image quality was systematically investigated. XRD patterns of Cs_2_Ag_0.6_Na_0.4_In_0.85_Bi_0.15_Cl_6_ (Supplementary Fig. S10) measured after long-term exposure to humid ambient air indicate its high structural stability, which outperforms that of the known lead-based perovskites. The irradiation stability and thermostability of Cs_2_Ag_0.6_Na_0.4_In_0.85_Bi_0.15_Cl_6_ under ambient air were further examined. As shown in Fig. 3e, the RL intensity shows no obvious degradation under thermal treatment for 50 h at 85 °C followed by continuous X-ray irradiation for another 50 h (Fig. 3e). The X-ray images of a circuit board acquired at three specific time points (0 h, 50 h and 100 h) can hardly be distinguished from each other (inset of Fig. 3e). In addition, the MTF values of the X-ray images acquired at 0 h, 50 h and 100 h show only a slight variation (Supplementary Fig. S11). All the above analyses provide evidence of the excellent feasibility of applying the Cs_2_Ag_0.6_Na_0.4_In_0.85_Bi_0.15_Cl_6_ scintillator in pervasive environments.

**Fig. 4:**
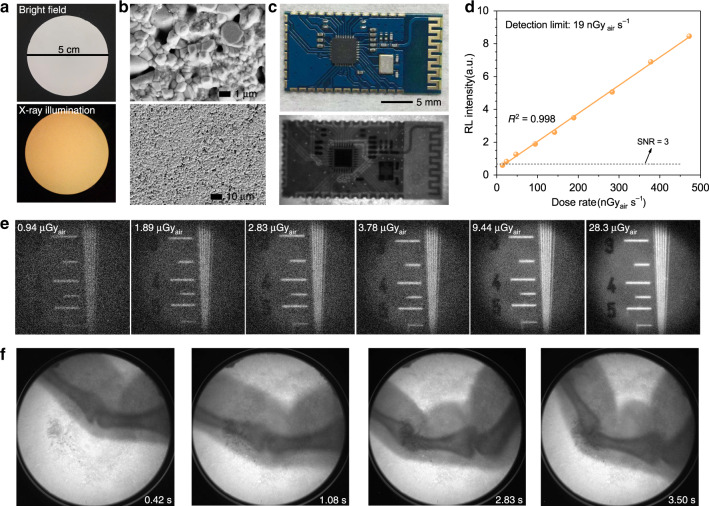
Real-time and low-dose X-ray imaging with Cs_2_Ag_0.6_Na_0.4_In_0.85_Bi_0.15_Cl_6_ scintillator wafers. **a** Photographs of a Cs_2_Ag_0.6_Na_0.4_In_0.85_Bi_0.15_Cl_6_ wafer under daylight and X-ray illumination (dose rate: 47.2 μGy_air_ s^−1^, voltage: 50 kV). **b** SEM images of the wafer surface. **c** Photograph of a circuit board (top) and its X-ray image (below) (dose rate: 47.2 μGy_air_ s^−1^, voltage: 50 kV). **d** RL intensity of Cs_2_Ag_0.6_Na_0.4_In_0.85_Bi_0.15_Cl_6_ measured at low-dose rates. The detection limit is derived from the fitting curve as the value when the SNR equals 3 (voltage: 50 kV). **e** X-ray images of the test-pattern plate acquired under different X-ray doses (voltage: 50 kV). **f** Real-time X-ray images of finger bending (dose rate: 47.2 μGy_air_ s^−1^, voltage: 50 kV)

Based on the optimised composition and thickness, a wafer of Cs_2_Ag_0.6_Na_0.4_In_0.85_Bi_0.15_Cl_6_ with a 0.1-mm thickness and a 5-cm diameter was prepared. SEM images of the wafer surface present its compactness and homogeneity (Fig. 4b). Utilising this wafer, we obtained X-ray images of the test-pattern plate under different doses of radiation (Fig. 4e), and the corresponding MTF results give resolutions ranging from 1.5 lp mm^−1^ to 4.4 lp mm^−1^ (Supplementary Fig. S12). This result demonstrates our scintillator’s capability to acquire high-resolution imaging under an extremely low dose of ∼1 μGy_air_, which is, to the best of our knowledge, the lowest dose requirement for perovskite-based X-ray imaging. The integrated RL intensity of Cs_2_Ag_0.6_Na_0.4_In_0.85_Bi_0.15_Cl_6_ measured in the low-dose rate range presents an excellent linear response to the X-ray dose rate (Fig. 4d). The detection limit derived from the fitting curve when the SNR equals 3 is 19 nGy_air_ s^−1^, which is much lower than the typical medical imaging dose. To assess the feasibility of using the Cs_2_Ag_0.6_Na_0.4_In_0.85_Bi_0.15_Cl_6_ scintillator for dynamic real-time X-ray imaging, a video of finger bending (Supplementary Video 1) was obtained under a low-dose rate of 47.2 μGy_air_ s^−1^, which exhibits a distinct phase contrast without a ghosting effect. The randomly selected X-ray images from the video at different time points show obvious biological tissue phase contrast and clear joint details (Fig. 4f). Figure 4c and Supplementary Fig. S13 are high-quality X-ray images of different circuit boards with various electronic components. An X-ray image of the complete test-pattern plate is shown in Supplementary Fig. S14. Considering these X-ray imaging demonstrations, the Cs_2_Ag_0.6_Na_0.4_In_0.85_Bi_0.15_Cl_6_ scintillator qualifies as a potential candidate for low-dose real-time X-ray imaging.

## Discussion

In conclusion, we developed a nontoxic Cs_2_Ag_0.6_Na_0.4_In_0.85_Bi_0.15_Cl_6_ double-perovskite scintillator, which exhibits not only a high light yield but also long-term stability under continuous thermal treatment and X-ray irradiation. High-resolution X-ray image attained under a low dose of ∼1 μGy_air_ and distinct real-time imaging of finger bending demonstrate its great potential for X-ray imaging technology. Our results reveal the huge potential in exploring scintillators beyond lead halide perovskites, not only for avoiding toxic elements but also for achieving higher performance.

## Methods

### Sample fabrication and characterisation

A series of Cs_2_Ag_1-x_Na_x_In_1-y_Bi_y_Cl_6_ single crystals were prepared by a slightly modified hydrothermal reaction^28^. First, 2 mmol of CsCl (99.9%, Xi’an Polymer Light Technology Corp.) was dissolved in 10 ml of a 10 M HCl (AR, Sinopharm) solution, followed by the addition of 1-x mol AgCl (99.999%, Aldrich), x mol NaCl (99.99%, Aladdin), 1-y mol InCl_3_ (99.999%, Aldrich) and y mol BiCl_3_ (99.99%, Macklin) at a certain ratio. The resulting mixture was transferred into a 50-ml Teflon autoclave and heated at 180 °C for 12 h. After cooling down to room temperature at 3 °C h^−1^, the precipitated millimetre-scale crystals were washed with isopropanol several times and dried at 60 °C in a vacuum oven.

The scintillator wafers were compressed from ground powder of the resulting bulk crystals using a hydraulic press. Approximately 50–500 mg of the powder was pressed at 15 MPa for 5 min to form scintillator wafers with thicknesses in the range of 0.1–1 mm. The thickness of the wafers was measured by a thickness gauge.

X-ray diffraction (XRD) analyses were carried out after grinding crystals into fine powder on an X-pert Powder diffractometer (PANalytical B.V.) with Cu-Kα radiation (*λ* = 0.15405 nm) in the 2*θ* range from 10° to 70°. Photoluminescence (PL) and PL excitation (PLE) spectra were recorded by an Edinburgh Instruments spectrofluorometer (FLS920). Scanning electron microscopy (SEM) images and energy-dispersive spectrometry (EDS) results were taken on a Hitachi SU8030 electron microscope equipped with an Oxford X-Max 20 silicon drift detector. Steady-state absorption spectra were detected using a home-built ultraviolet–visible spectrophotometer system equipped with an integrating sphere. PL quantum yield (PLQY) measurements were performed using an absolute photoluminescence measurement system (Hamamatsu Quantaurus-QY). Time-resolved photoluminescence (TRPL) decay kinetics were collected using a time-correlated single photon counting (TCSPC) module (PicoHarp 300) and an SPAD detector (IDQ, id100). The sample was excited by a femtosecond laser (Light Conversion Pharos, 400 nm, <300 fs, 1 MHz). Afterglow curves were detected using the single shot transient digitiser (SSTD) technique. Radioluminescence (RL) spectra were measured by a fibre-coupled fluorescence spectrometer (Ocean Optics QE PRO) equipped with an integrating sphere and a fixed X-ray source-to-sample distance.

### Light-yield measurement

As is known, the light yield can be regarded as the ratio of the number of photons emitted from the luminescent sites to the total absorbed X-ray energy. First, we separately calculated the attenuation efficiency of LuAG:Ce (22,000 ± 4000 photons/MeV) and Cs_2_Ag_0.6_Na_0.4_In_0.85_Bi_0.15_Cl_6_ as a function of sample thickness at an X-ray photon energy of 22 keV. To unify the absorbed X-ray energies of these two kinds of scintillator, a Cs_2_Ag_0.6_Na_0.4_In_0.85_Bi_0.15_Cl_6_ wafer with a 0.4-mm thickness and a LuAG:Ce wafer with a 0.11-mm thickness were compressed. Then, the scintillator wafers were closely attached to the circular window of an integrating sphere with a fixed distance to the X-ray source, and the corresponding RL spectra were recorded by a QE PRO fibre-coupled fluorescence spectrometer (carefully calibrated by an Ocean Optics engineer before usage). Comparing the integrated intensities of the two spectra, a light yield of 39,000 ± 7000 photons/MeV for the Cs_2_Ag_0.6_Na_0.4_In_0.85_Bi_0.15_Cl_6_ scintillator was acquired. The measurement system was cross-checked with another commercial scintillator of CsI:TI, obtaining a light yield of 57,000 photons/MeV, which matches its datasheet value (~60,000 photons/MeV) and proves the validity of the measurement method.

### X-ray imaging optical system setup

A Mini-X X-ray tube (target material: Ag, *P*_max_ = 4 W, *V*_max_ = 50 kV, *I*_max_ = 79 μA) produced by Amptek Inc. was utilised as the X-ray source, generating an X-ray output spectrum with both intense characteristic radiation of Ag and broad bremsstrahlung radiation. The average X-ray photon energy was ~22 keV. The X-ray dose rates were altered by adjusting the current of the X-ray tube from 5 μA to 79 μA, and were calibrated by a highly sensitive X-ray ion chamber dose meter (Radcal Corporation 10 × 5-180). The objects and scintillator wafers were placed vertically to the incident X-rays, and the scintillators were fixed just behind the objects. A reflector was utilised to deflect the optical path by 90° to diminish the negative influence caused by direct radiation from the X-ray source on the camera. To collect X-ray images, a CMOS camera (Photometrics 95B) with 1200 × 1200 pixels and a 11-μm pixel size was equipped.

### MTF measurements

MTF represents the capability to transfer the input signal modulation at a given spatial frequency to its output, and can be used to evaluate the fundamental spatial resolution performance of an imaging system. The spatial resolution can be determined by the spatial frequency value when MTF = 0.2. The MTF curve was calculated by the slanted-edge method. First, a piece of aluminium (thickness: ~1 mm) with a sharp edge was placed on the scintillator, and its edge profile was derived from the resulting X-ray image. Then, the edge spread function (ESF) was derived from the edge profile, from which we could deduce the line spread function (LSF). Finally, the MTF values were defined by the Fourier transform of the LSF as follows:

$${\it{{\mathrm{MTF}}}}\left( {\it{\upnu }} \right) = {\it{F}}\left( {{\it{{\mathrm{LSF}}}}\left( {\it{x}} \right)} \right) = {\it{F}}\left( {\frac{{{\it{{\mathrm{dESF}}}}\left( {\it{x}} \right)}}{{{\it{{\mathrm{d}}x}}}}} \right),$$

where *ν* is the spatial frequency, and *x* is the position of the pixels.

## Supplementary information

Supplementary Information

Supplementary Information

Supplementary Information

Supplementary Information

Supplementary Information
