# Supplementary material for: Low-dose real-time X-ray imaging with nontoxic double perovskite scintillators
Source: Light Sci Appl. 2020 Jun 30;9:112. doi: 10.1038/s41377-020-00353-0 (PMC7327019; doi:10.1038/s41377-020-00353-0)
Supplement: Supplementary file 1 — Supplementary Information [file 41377_2020_353_MOESM1_ESM.docx]

**Supplementary information for:**

**Low-Dose Real-Time X-ray Imaging with Nontoxic Double Perovskite Scintillators**

Wenjuan Zhu^1,3^, Wenbo Ma^1,3^, Yirong Su^1^, Zeng Chen^2^, Xinya Chen^1^, Yaoguang Ma^1^, Lizhong Bai^1^, Wenge Xiao^1^, Tianyu Liu^1^, Haiming Zhu^2^, Xiaofeng Liu^1^, Huafeng Liu^1^, Xu Liu^1^, and Yang (Michael) Yang^1^*

^1^State Key Laboratory of Modern Optical Instrumentation, College of Optical Science and Engineering, Zhejiang University, Hangzhou, Zhejiang, China.

^2^Center for Chemistry of High-Performance & Novel Materials, department of Chemistry, Zhejiang University, Hangzhou, Zhejiang, China.

^3^These authors contributed equally: Wenjuan Zhu, Wenbo Ma.

*e-mail: [yangyang15@zju.edu.cn](mailto:yangyang15@zju.edu.cn);


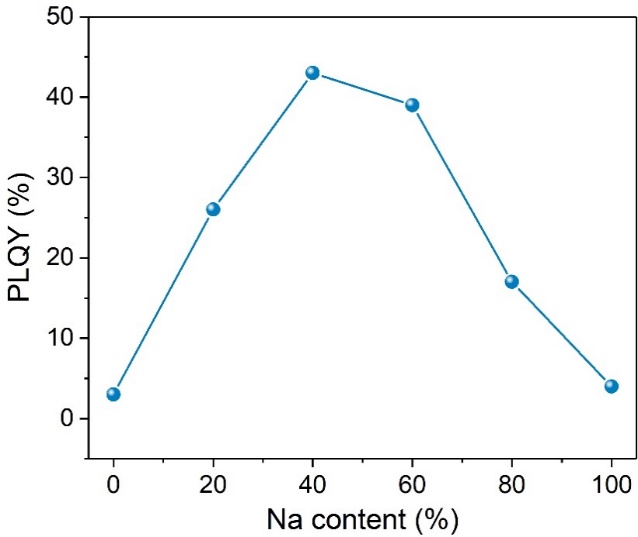




**Fig. S1 |** PLQY (left) and XRD patterns (right) of Cs_2_Ag_1-x_Na_x_InCl_6_ powder with various Na^+^ contents.


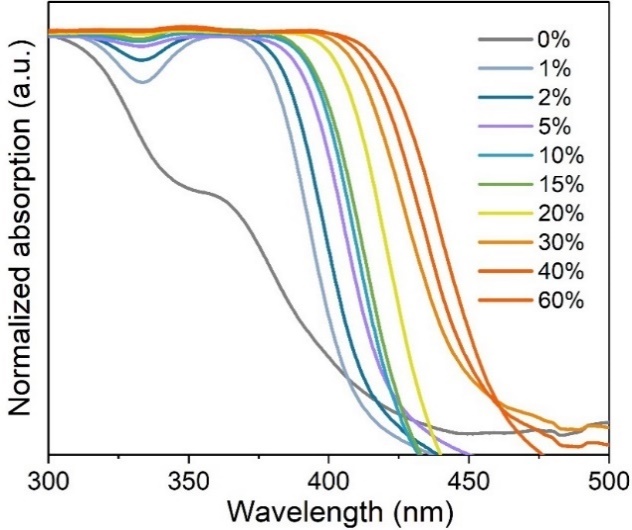




**Fig. S2 |** Normalized steady-state absorption spectra (left) and plots of absorption as a function of photon energy to extract Urbach energy E_U_ (right) of Cs_2_Ag_0.6_Na_0.4_In_1-y_Bi_y_Cl_6_ with different Bi^3+^ contents.







**Fig. S3 |** Tauc plots (left) and bandgap energy (right) for Cs_2_Ag_0.6_Na_0.4_In_1-y_Bi_y_Cl_6_ samples with different Bi^3+^ contents.


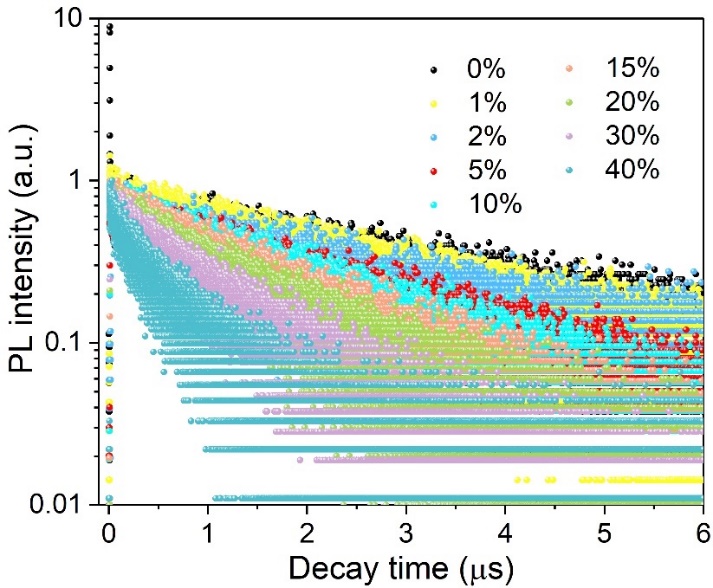

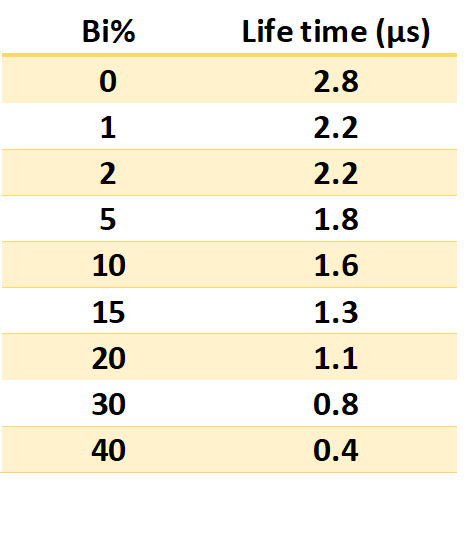


**Fig. S4 |** TRPL decay spectra monitored at the wavelength range from 407 nm to 800 nm (left) and corresponding lifetime values of the slower decay process (right) for Cs_2_Ag_0.6_Na_0.4_In_1-y_Bi_y_Cl_6_ samples with different Bi^3+^ contents.

**
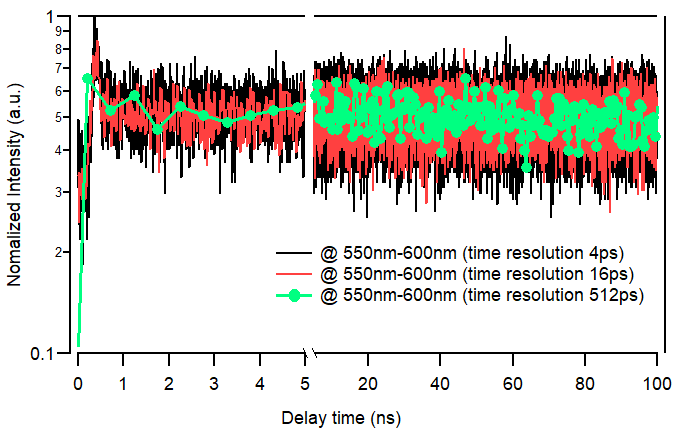

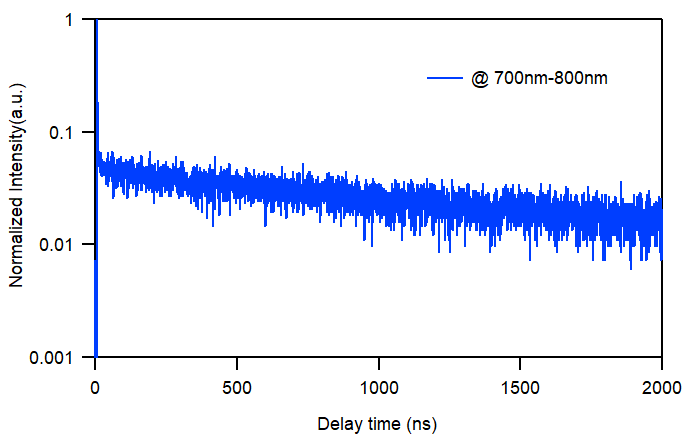
**

**Fig. S5 |** TRPL decay spectra of Cs_2_Ag_0.6_Na_0.4_InCl_6_ detected at different time resolution (4 ps/16 ps/512 ps) (left), and monitored at the wavelength range from 700 nm to 800 nm (right).


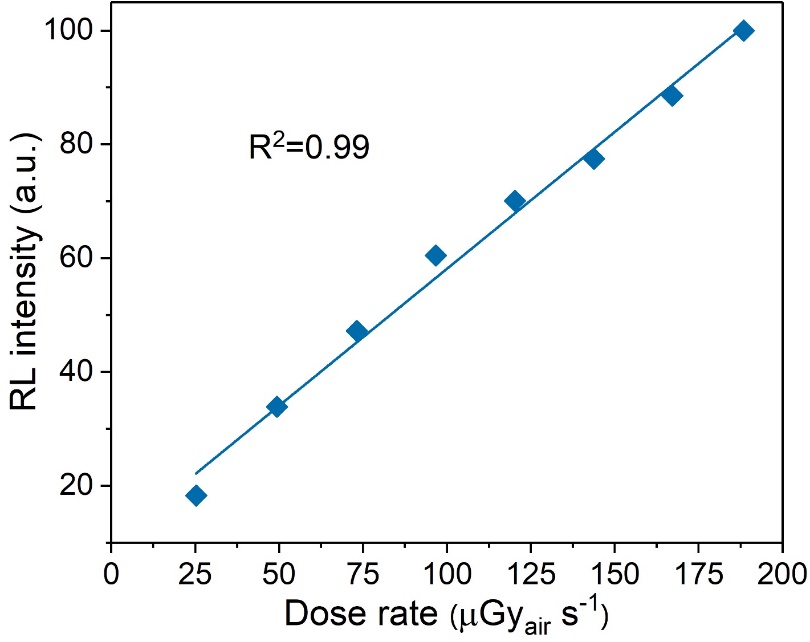


**Fig. S6 |** RL intensity of Cs_2_Ag_0.6_Na_0.4_In_0.85_Bi_0.15_Cl_6_ powder as a function of X-ray dose rate (voltage: 50 kV)





**Fig. S7 |** Attenuation efficiency of Cs_2_Ag_0.6_Na_0.4_In_1-y_Bi_y_Cl_6_, LuAG: Ce and CsI: Tl as a function of thickness.





**Fig. S8 |** Attenuation efficiency of Cs_2_Ag_0.6_Na_0.4_In_0.85_Bi_0.15_Cl_6_, LuAG:Ce and CsI:Tl as a function of photon energy.


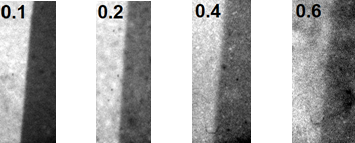


**Fig. S9 |** X-ray slanted-edge images of Cs_2_Ag_0.6_Na_0.4_In_0.85_Bi_0.15_Cl_6_ wafers with different thicknesses (0.1 mm, 0.2 mm, 0.4 mm and 0.6 mm).


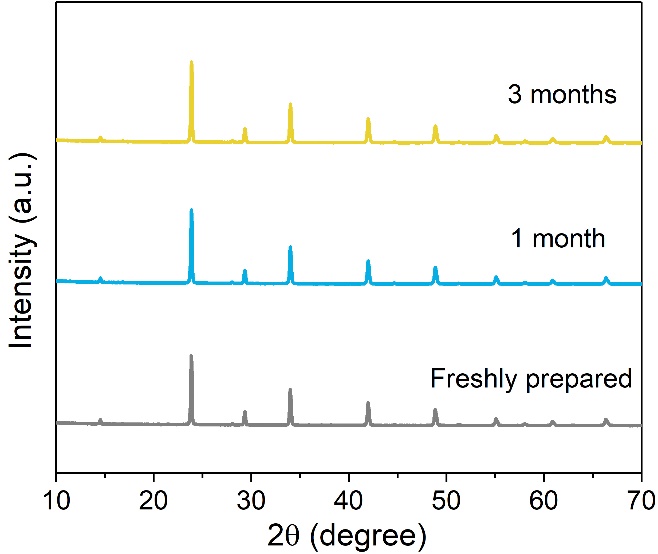


**Fig. S10 |** XRD patterns of Cs_2_Ag_0.6_Na_0.4_In_0.85_Bi_0.15_Cl_6_ measured after long-term exposure to ambient air.


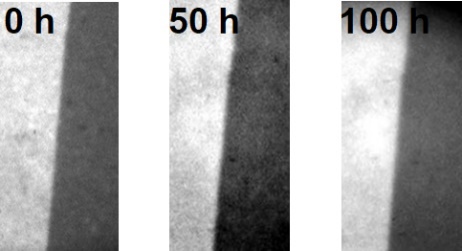

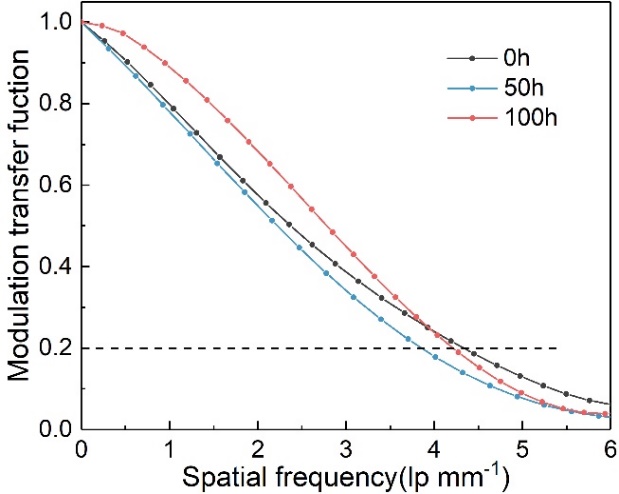


**Fig. S11 |** X-ray slanted-edge images (left) and the corresponding MTF curves (right) acquired at three different stages (0 h, 50 h and 100 h).


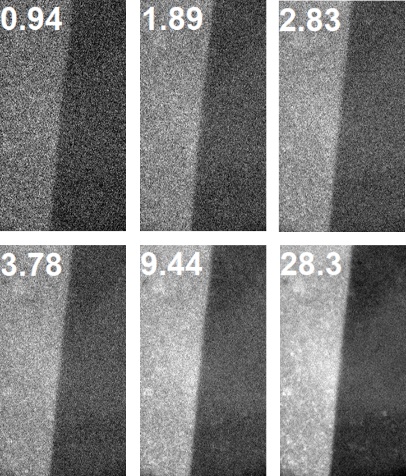

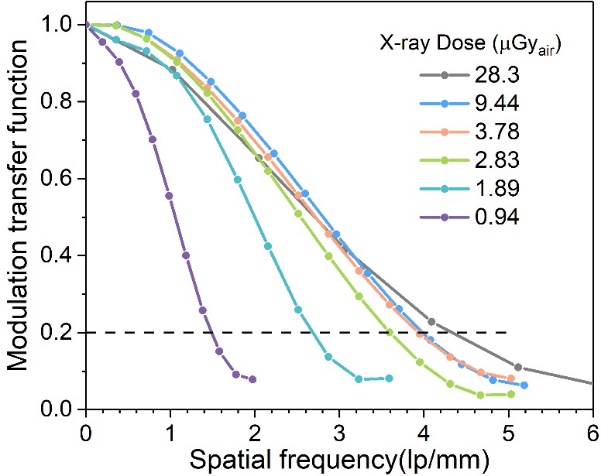


**Fig. S12 |** X-ray slanted-edge images (left) and the corresponding MTF curves (right) acquired from different X-ray doses (unti: μGy_air_).


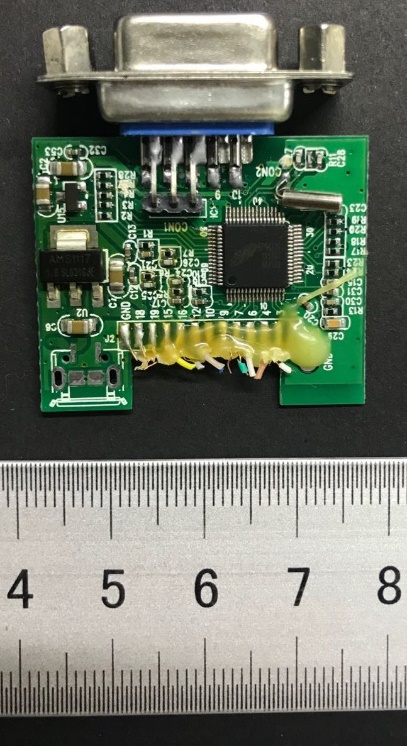

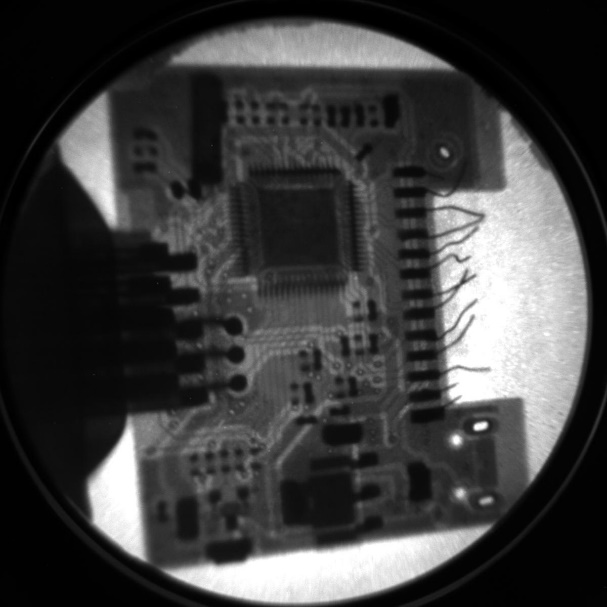


**Fig. S13 |** Photograph of a circuit board (left) and its X-ray image (right) (dose rate: 47.2 μGy_air_ s^-1^， voltage: 50 kV ).


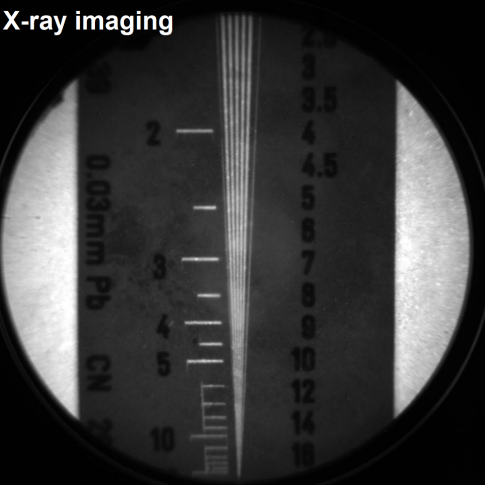


**Fig. S14 |** X-ray image of full test pattern plate. (dose rate: 47.2 μGy_air_ s^-1^, voltage: 50 kV)

**Table S1 |** Afterglow data of the Cs_2_Ag_0.6_Na_0.4_In_0.85_Bi_0.15_Cl_6_ and some commercial scintillators.


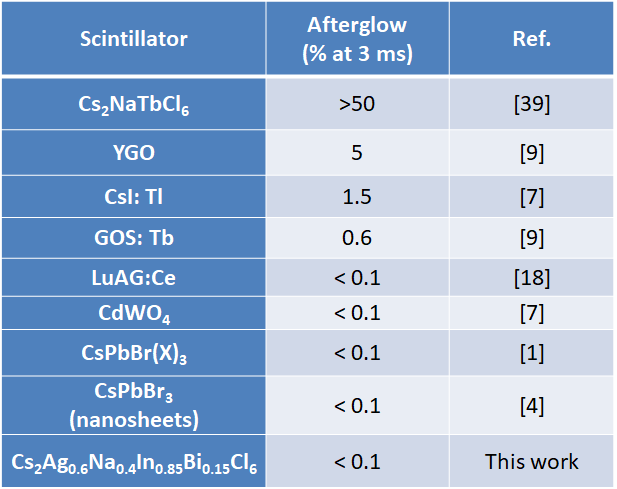


**Table S2 |** The SEM-EDS results of Cs_2_Ag_0.6_Na_0.4_In_1-x_Bi_x_Cl_6_ with different Bi^3+^ contents.

| x=0% | No. | Cs | Na | Ag | In | Bi | Cl |
| --- | --- | --- | --- | --- | --- | --- | --- |
|  | 1 | 19.58 | 4.16 | 5.4 | 9.78 | 0 | 61.08 |
|  | 2 | 19.87 | 3.56 | 5.3 | 9.59 | 0 | 61.68 |
|  | 3 | 20.00 | 3.42 | 5.47 | 9.63 | 0 | 61.48 |
|  | 4 | 19.91 | 3.33 | 5.64 | 9.71 | 0 | 61.42 |
|  | 5 | 19.35 | 3.76 | 6.02 | 9.67 | 0 | 61.2 |
|  | 6 | 19.7 | 3.21 | 5.8 | 9.99 | 0 | 61.3 |
|  | Average | 19.74 | 3.57 | 5.60 | 9.73 | 0 | 61.36 |

| x=1% | No. | Cs | Na | Ag | In | Bi | Cl |
| --- | --- | --- | --- | --- | --- | --- | --- |
|  | 1 | 19.43 | 4.28 | 5.38 | 9.82 | 0.15 | 60.95 |
|  | 2 | 19.34 | 4.03 | 5.58 | 9.81 | 0.05 | 61.19 |
|  | 3 | 18.92 | 3.93 | 5.94 | 9.75 | 0.15 | 61.32 |
|  | 4 | 19.4 | 3.73 | 5.95 | 9.72 | 0.17 | 61.03 |
|  | 5 | 19.14 | 4.94 | 5.69 | 9.54 | 0.05 | 60.64 |
|  | 6 | 19.52 | 3.62 | 5.99 | 9.51 | 0.10 | 61.26 |
|  | Average | 19.29 | 4.09 | 5.76 | 9.69 | 0.11 | 61.06 |

| x=2% | No. | Cs | Na | Ag | In | Bi | Cl |
| --- | --- | --- | --- | --- | --- | --- | --- |
|  | 1 | 19.48 | 4.14 | 5.22 | 9.57 | 0.18 | 61.41 |
|  | 2 | 19.62 | 3.05 | 6.00 | 9.50 | 0.18 | 61.65 |
|  | 3 | 19.95 | 3.98 | 5.17 | 9.85 | 0.28 | 60.77 |
|  | 4 | 19.7 | 3.46 | 5.19 | 9.87 | 0.19 | 61.59 |
|  | 5 | 19.15 | 4.66 | 5.4 | 9.46 | 0.27 | 61.04 |
|  | 6 | 19.37 | 4.25 | 5.32 | 9.67 | 0.2 | 61.18 |
|  | Average | 19.55 | 3.92 | 5.39 | 9.65 | 0.22 | 61.27 |

| x=5% | No. | Cs | Na | Ag | In | Bi | Cl |
| --- | --- | --- | --- | --- | --- | --- | --- |
|  | 1 | 19.5 | 3.66 | 5.75 | 9.44 | 0.45 | 61.19 |
|  | 2 | 19.59 | 4.54 | 5.58 | 8.91 | 0.48 | 60.90 |
|  | 3 | 19.67 | 4.37 | 5.21 | 9.18 | 0.48 | 61.09 |
|  | 4 | 19.42 | 3.42 | 6.29 | 9.1 | 0.6 | 61.18 |
|  | 5 | 20.43 | 4.07 | 5.46 | 8.73 | 0.44 | 60.87 |
|  | 6 | 20.49 | 4.02 | 5.14 | 8.96 | 0.6 | 60.79 |
|  | Average | 19.85 | 4.01 | 5.57 | 9.06 | 0.51 | 61.00 |

| x=10% | No. | Cs | Na | Ag | In | Bi | Cl |
| --- | --- | --- | --- | --- | --- | --- | --- |
|  | 1 | 19.3 | 4.13 | 5.4 | 9.07 | 0.89 | 61.22 |
|  | 2 | 20.66 | 3.6 | 5.26 | 8.52 | 1.12 | 60.84 |
|  | 3 | 19.49 | 4.01 | 5.43 | 8.64 | 1.00 | 61.43 |
|  | 4 | 19.8 | 3.19 | 6.04 | 9.01 | 0.76 | 61.20 |
|  | 5 | 19.1 | 4.82 | 5.66 | 8.58 | 0.96 | 60.88 |
|  | 6 | 19.53 | 4.05 | 5.51 | 8.53 | 1.15 | 61.22 |
|  | Average | 19.65 | 3.97 | 5.55 | 8.72 | 0.98 | 61.13 |

| x=15% | No. | Cs | Na | Ag | In | Bi | Cl |
| --- | --- | --- | --- | --- | --- | --- | --- |
|  | 1 | 19.75 | 4.31 | 5.73 | 8.23 | 1.56 | 60.42 |
|  | 2 | 19.27 | 2.94 | 6.26 | 8.26 | 1.48 | 61.79 |
|  | 3 | 18.98 | 4.87 | 6.04 | 8.15 | 1.6 | 60.35 |
|  | 4 | 20.22 | 2.66 | 6.47 | 8.15 | 1.50 | 61 |
|  | 5 | 19.31 | 3.77 | 6.19 | 8.04 | 1.55 | 61.14 |
|  | 6 | 19.3 | 4.51 | 5.58 | 8.17 | 1.46 | 60.98 |
|  | Average | 19.47 | 3.84 | 6.04 | 8.17 | 1.53 | 60.95 |

| x=20% | No. | Cs | Na | Ag | In | Bi | Cl |
| --- | --- | --- | --- | --- | --- | --- | --- |
|  | 1 | 19.31 | 4.68 | 4.88 | 8.07 | 1.79 | 61.28 |
|  | 2 | 20.16 | 4.15 | 5.08 | 7.92 | 2.1 | 60.59 |
|  | 3 | 18.92 | 3.84 | 7.06 | 7.9 | 2.17 | 60.11 |
|  | 4 | 19.18 | 4.71 | 6.02 | 8.4 | 1.74 | 59.95 |
|  | 5 | 20.8 | 4.96 | 4.82 | 7.69 | 1.94 | 59.79 |
|  | 6 | 19.77 | 3.09 | 6.79 | 7.95 | 1.56 | 60.84 |
|  | Average | 19.69 | 4.24 | 5.77 | 7.99 | 1.88 | 60.43 |

| x=30% | No. | Cs | Na | Ag | In | Bi | Cl |
| --- | --- | --- | --- | --- | --- | --- | --- |
|  | 1 | 18.68 | 4.2 | 6.64 | 7.44 | 2.61 | 60.44 |
|  | 2 | 19.26 | 3.75 | 5.35 | 7.38 | 2.79 | 61.48 |
|  | 3 | 19.88 | 2.45 | 7.14 | 6.74 | 3.31 | 60.48 |
|  | 4 | 19.75 | 2.49 | 7.32 | 6.52 | 3.14 | 60.77 |
|  | 5 | 19.53 | 4.42 | 4.63 | 7.70 | 2.82 | 60.91 |
|  | 6 | 19.79 | 4.72 | 5.12 | 6.51 | 2.69 | 61.17 |
|  | Average | 19.48 | 3.67 | 6.033 | 7.05 | 2.89 | 60.88 |

| x=40% | No. | Cs | Na | Ag | In | Bi | Cl |
| --- | --- | --- | --- | --- | --- | --- | --- |
|  | 1 | 20.86 | 4.12 | 4.68 | 6.28 | 4.14 | 59.92 |
|  | 2 | 19.85 | 3.38 | 6.26 | 6 | 3.78 | 60.74 |
|  | 3 | 19.32 | 2.91 | 7 | 6.43 | 3.66 | 60.67 |
|  | 4 | 20.03 | 3.21 | 6.08 | 6.27 | 3.87 | 60.55 |
|  | 5 | 18.79 | 4.37 | 5.57 | 6.28 | 4.02 | 60.96 |
|  | 6 | 18.63 | 5.21 | 5.04 | 6.14 | 3.78 | 61.2 |
|  | Average | 19.58 | 3.87 | 5.77 | 6.23 | 3.88 | 60.67 |

| x=60% | No. | Cs | Na | Ag | In | Bi | Cl |
| --- | --- | --- | --- | --- | --- | --- | --- |
|  | 1 | 22.72 | 2.79 | 6.59 | 4.36 | 5.75 | 57.80 |
|  | 2 | 20.96 | 4.05 | 6.14 | 3.72 | 6.12 | 59.01 |
|  | 3 | 20.11 | 3.65 | 6.19 | 4.16 | 6.39 | 59.5 |
|  | 4 | 20.77 | 3.02 | 6.59 | 3.85 | 6.27 | 59.5 |
|  | 5 | 19.3 | 3.04 | 7.25 | 4.89 | 5.59 | 59.93 |
|  | 6 | 19.45 | 3.59 | 6.72 | 4.8 | 5.56 | 59.89 |
|  | Average | 20.55 | 3.36 | 6.58 | 4.30 | 5.95 | 59.27 |

x=0%


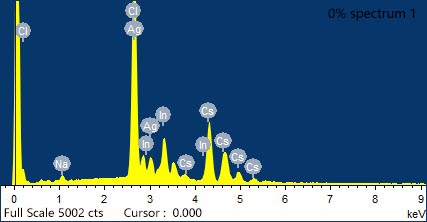

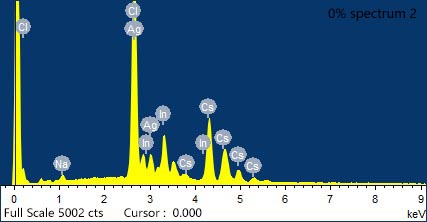


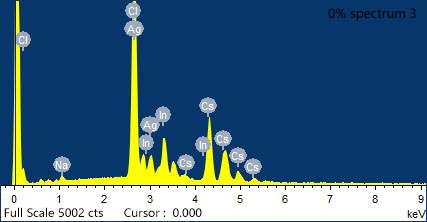

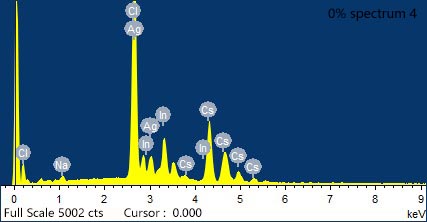


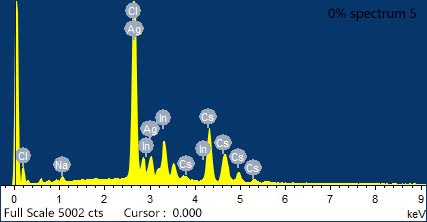

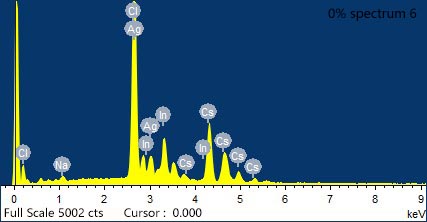


x=1%


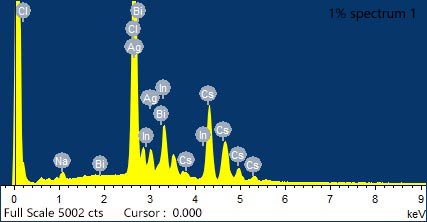

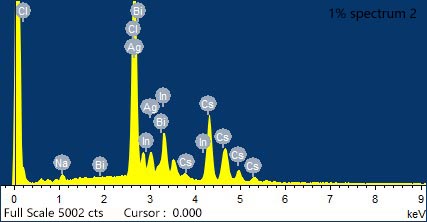


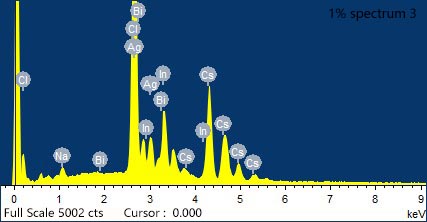

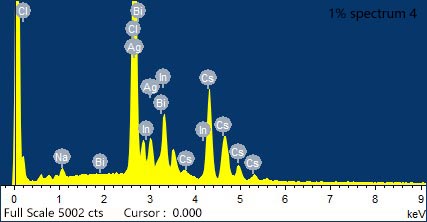


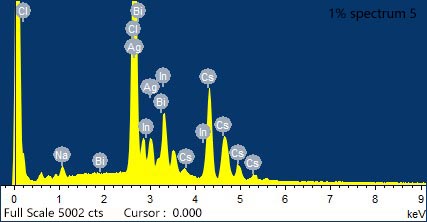

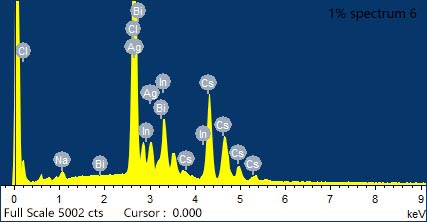


x=2%


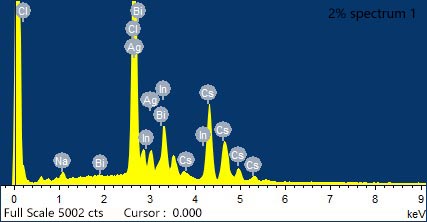

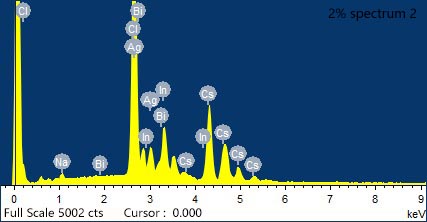


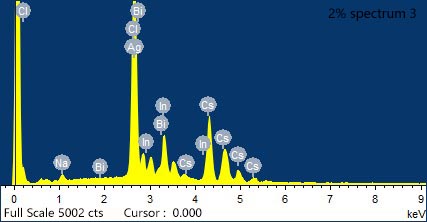

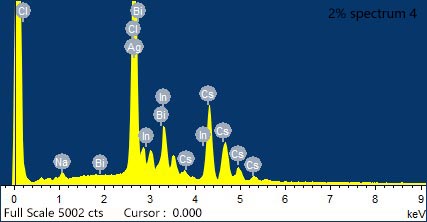


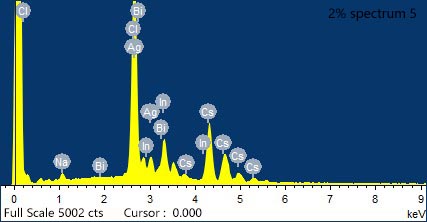

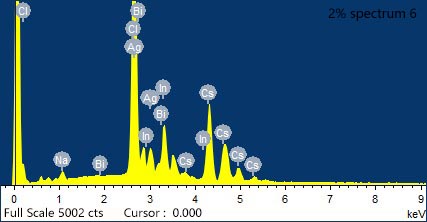


x=5%


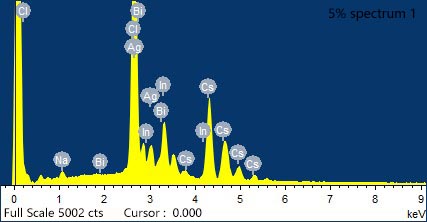

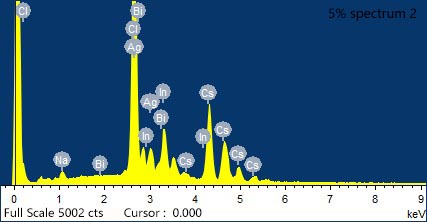


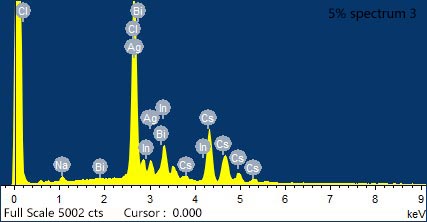

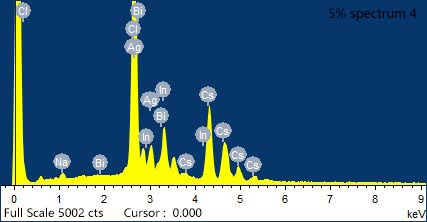


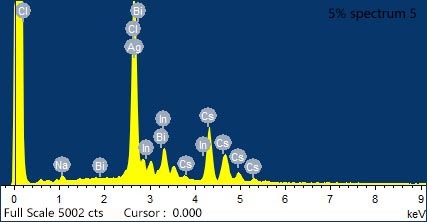

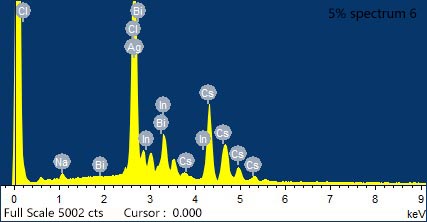


x=10%


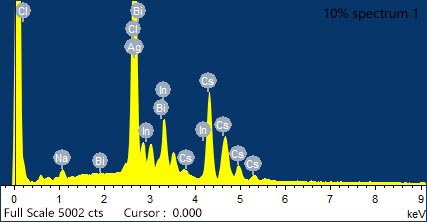

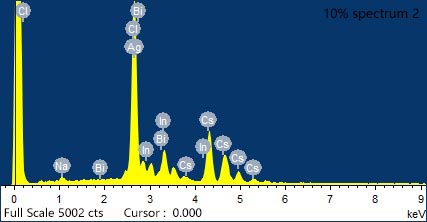


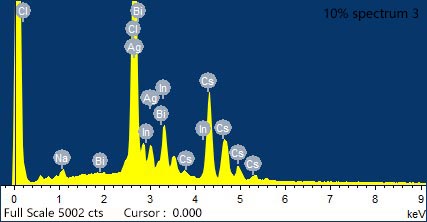

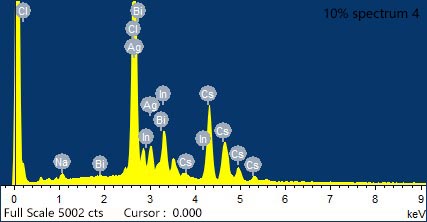


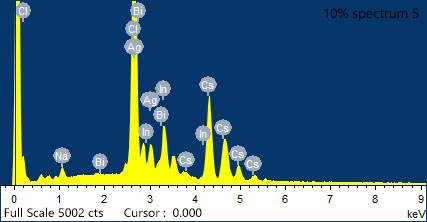

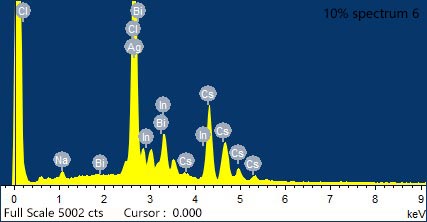


x=15%


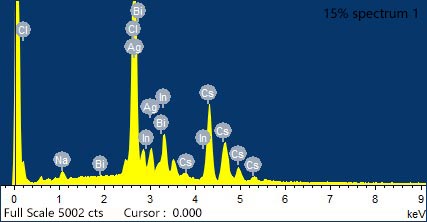

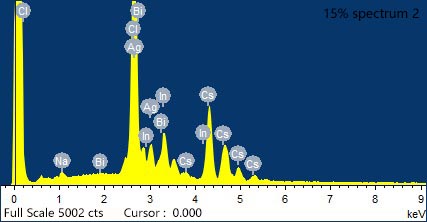


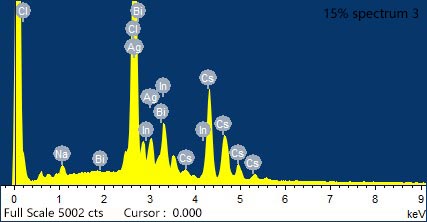

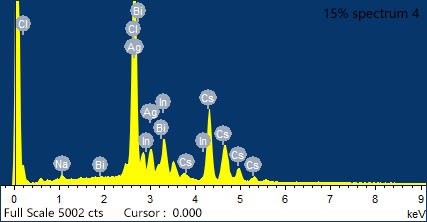


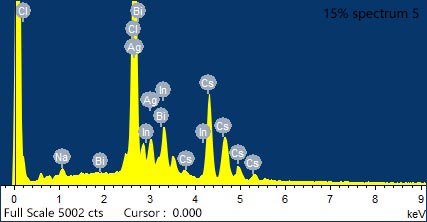

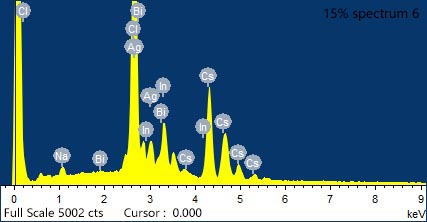


x=20%


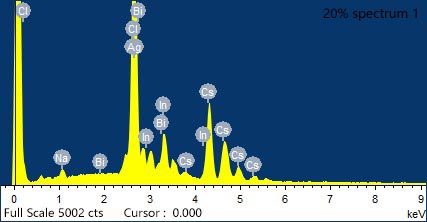

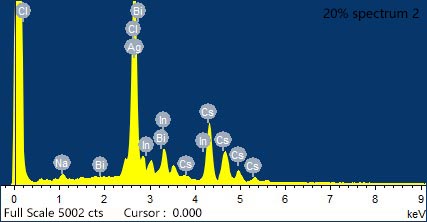


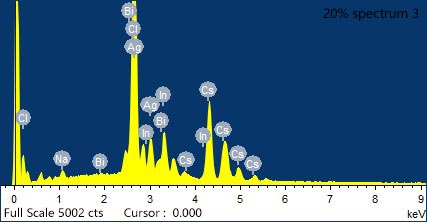

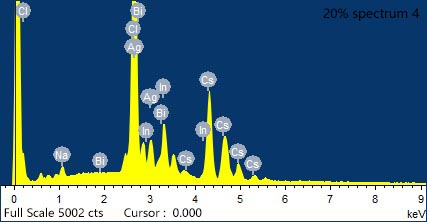


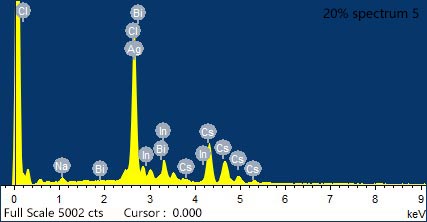

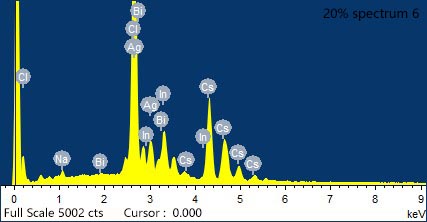


x=30%


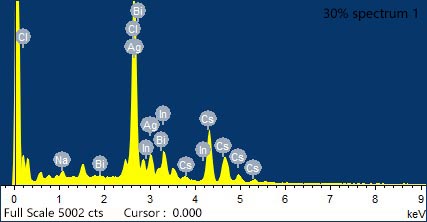

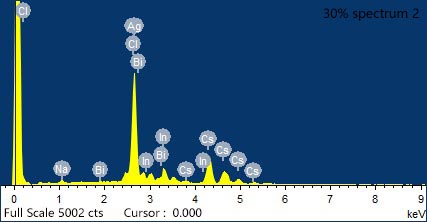


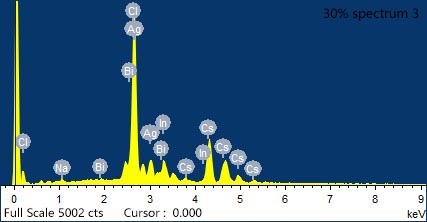

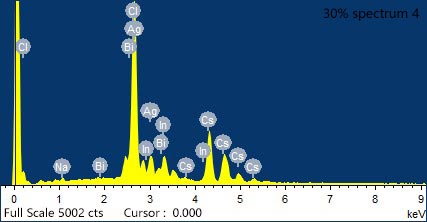


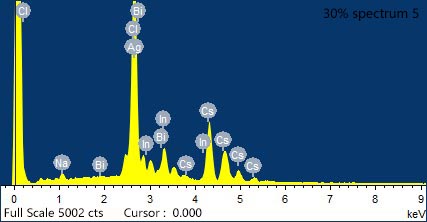

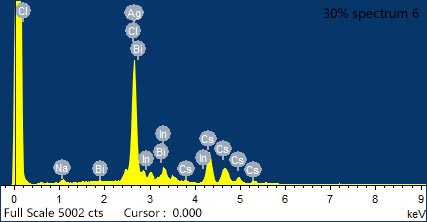


x=40%


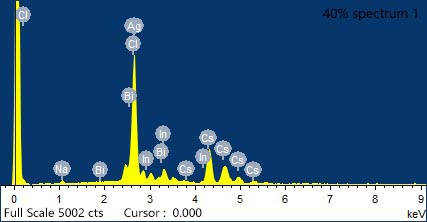

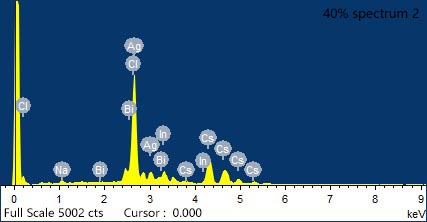

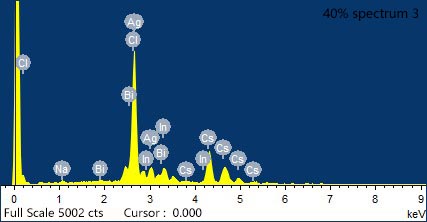

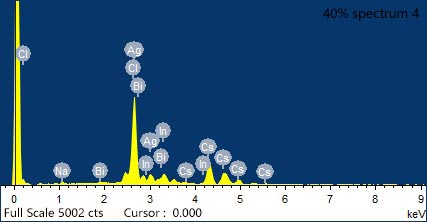


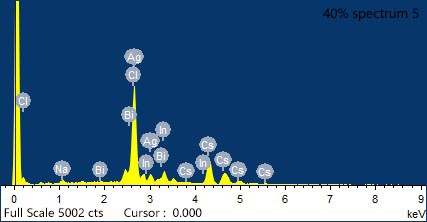

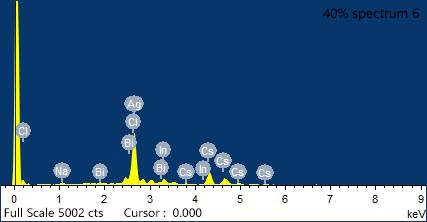


x=60%


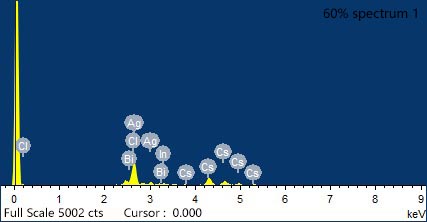

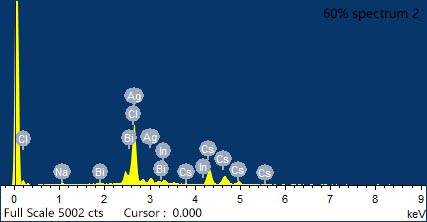

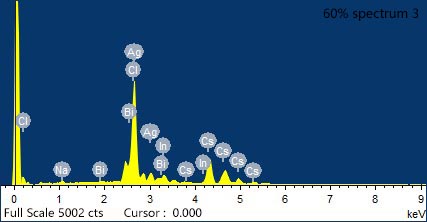

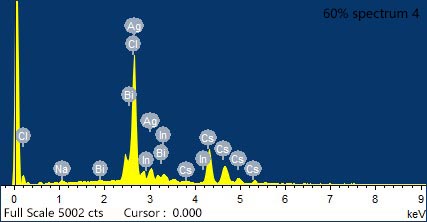


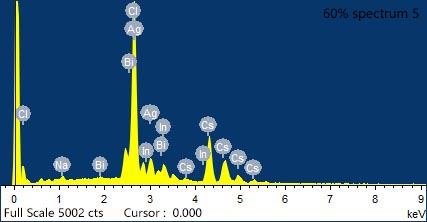

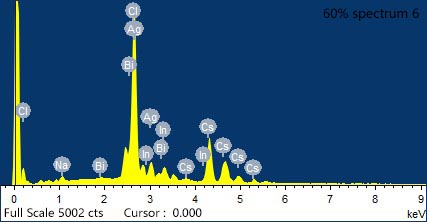


**Fig. S15 |** The SEM-EDS spectra of Cs_2_Ag_0.6_Na_0.4_In_1-x_Bi_x_Cl_6_ with different Bi^3+^ contents.
